# Self-Sustained Motor Activity Triggered by Interlimb Reflexes in Chronic Spinal Cord Injury, Evidence of Functional Ascending Propriospinal Pathways

**DOI:** 10.1371/journal.pone.0072725

**Published:** 2013-08-02

**Authors:** Penelope A. McNulty, David Burke

**Affiliations:** 1 Neuroscience Research Australia and University of New South Wales, Sydney, Australia; 2 Institute of Clinical Neurosciences, Royal Prince Alfred Hospital, and University of Sydney, Sydney, Australia; Hospital Nacional de Parapléjicos, Spain

## Abstract

The loss or reduction of supraspinal inputs after spinal cord injury provides a unique opportunity to examine the plasticity of neural pathways within the spinal cord. In a series of nine experiments on a patient, quadriplegic due to spinal cord injury, we investigated interlimb reflexes and self-sustained activity in completely paralyzed and paretic muscles due to a disinhibited propriospinal pathway. Electrical stimuli were delivered over the left common peroneal nerve at the fibular head as single stimuli or in trains at 2–100 Hz lasting 1 s. Single stimuli produced a robust interlimb reflex twitch in the contralateral thumb at a mean latency 69 ms, but no activity in other muscles. With stimulus trains the thumb twitch occurred at variable subharmonics of the stimulus rate, and strong self-sustained activity developed in the contralateral wrist extensors, outlasting both the stimuli and the thumb reflex by up to 20 s. Similar behavior was recorded in the ipsilateral wrist extensors and quadriceps femoris of both legs, but not in the contralateral thenar or peroneal muscles. The patient could not terminate the self-sustained activity voluntarily, but it was abolished on the left by attempted contractions of the paralyzed thumb muscles of the right hand. These responses depend on the functional integrity of an ascending propriospinal pathway, and highlight the plasticity of spinal circuitry following spinal cord injury. They emphasize the potential for pathways below the level of injury to generate movement, and the role of self-sustained reflex activity in the sequelae of spinal cord injury.

## Introduction

In human bipedal locomotion the arm swing is thought to be a vestigial remnant of quadrupedal gait patterns [1]. This limb co-ordination occurs through the linking via propriospinal pathways of caudal central pattern generators that control hindlimb movement with central pattern generators in the rostral spinal cord controlling forelimb movement [2]. Long propriospinal connections mediate interlimb cutaneomuscular reflexes in healthy human subjects [3,4], although these have been demonstrable only during voluntary contractions of the target muscle. They may provide interlimb co-ordination for locomotion [4] or protection against limb trauma [3]. There is abundant evidence for descending propriospinal pathways in humans with modulation of the soleus H reflex by upper limb stimulation [5] or continuous movement [6,7]. There is less evidence for functional ascending propriospinal pathways which have been demonstrable only after potentiation through high-frequency stimulation [4] or with maximal voluntary efforts [8]. Studies reporting bidirectional propriospinal activation show that the descending pathway is easier to demonstrate than the ascending pathway [4,8,9], and that the ascending pathway is stronger ipsilaterally than contralaterally [8,9].

Propriospinal connections can undergo regenerative sprouting after spinal cord injury (SCI) [10] and presumably mediate phase-dependent modulation of lower-limb EMG activity with passive upper-limb movement in patients with SCI [11]. Changes in segmental circuits after SCI can be sufficient to trigger myoclonus [12], sustained activity causing rigidity [13] and flexor spasms, hyperreflexia and hypertonia [14,15].

Self-sustained motor activity (activity that once triggered may continue long after the stimulus) has been documented for single motor units [16] and whole muscles [17] in healthy human subjects and in patients following stroke [18] and SCI [19]. However there has been only a single example of self-sustained activity generated over an interlimb circuit, and that occurred following SCI [20]. This activity was set up by small myelinated thermal (Aδ) afferents: there are no reports of such activity triggered by large tactile afferents in SCI. Self-sustained activity has generally been attributed to plateau potentials in motoneurones, driven by persistent inward currents (PICs) involving Na^+^ channels and L-type Ca^2+^ channels [21,22], and may contribute to the clinical features of, particularly, SCI [18,21–24]. Interestingly the trigger for this activity has generally involved muscle afferents.

The present study provides strong evidence for a long ascending propriospinal pathway in humans mediated by low-threshold cutaneous afferents. The dominant response was to the contralateral upper limb, although responses were seen in all four limbs. Our detailed study of this pathway demonstrates the potential for self-sustained activity to contribute to the pathophysiology of SCI and highlights the plasticity of spinal cord circuitry following SCI.

## Methods

Nine experiments were performed over 18 months on a 39 year old female, C5-6 quadriplegic, ASIA A, following a motor vehicle accident 20 years earlier, patient A. Due to a decubitus ulcer, a left sciatic nerve palsy had developed >5 years prior to testing. This caused inexcitability of the left peroneal and tibial nerves, even in response to stimulus intensities of 200 mA. A left lumbar sympathectomy had been performed 4 years earlier for neuropathic pain. The subject gave signed, informed consent to the experimental procedures if non-invasive, but declined invasive studies. All experiments were approved by the Human Research Ethics Committee of the University of New South Wales and conducted in accordance with the Declaration of Helsinki.

On neurological examination the subject had a clinically complete spinal cord lesion below C6 bilaterally. There was weak shoulder abduction, elbow flexion and wrist extension, but wrist flexors, triceps brachii and the intrinsic muscles of the hand were completely paralyzed to clinical examination and using EMG recordings, as were abdominal and lower limb muscles. These findings were confirmed using transcranial magnetic stimulation of the motor cortex (see Results). The subject experienced involuntary spasms with posture-dependent trunk and abdominal spasms. Sensation was normal above C4, incomplete and hyperesthetic at C6 and completely absent below that level. She had an upper motoneuron bladder and used a suprapubic catheter. Although she originally required medication for the “spastic” bladder, she had not taken specific medication for spasticity or bladder issues for >10 years. The patient experienced autonomic dysreflexia, but this did not occur during experimental sessions. During experiments, the subject sat in her wheelchair with the ankle, knee and hip flexed as close to 90^°^ as possible. Her upper limbs were not restrained due to spasms.

The observations reported here arose from an investigation of motor unit activity in tibialis anterior after spinal cord injury (unpublished data). Eighteen patients with spinal cord injury have been investigated, 10 bilaterally, using percutaneous electrical stimulation at ≤1 Hz applied to the common peroneal nerve at the fibular head. The patients were aged from 22 to 54 years of age, with injuries from C3 to T12 and 40 healthy controls were aged from 18 to 49 years. Single motor unit responses were studied in a further 10 spinal cord injury subjects using the same constant current stimulus trains from 2 to 100 Hz. These patients were 31 to 63 years with injuries from C5 to T12. We saw no evidence of interlimb reflexes or self-sustained activity in these patients or healthy control subjects using these stimulus frequencies or intensities.

A detailed search for ascending propriospinally mediated inter-limb reflex activity was made using the full protocol reported here in an additional two patients with a spinal cord injury and in two healthy control subjects, a female aged 44 and a male aged 36. Patient B was a 44 year old male with a 9 year C5-6 injury with weak shoulder abduction, elbow flexion and wrist extension, no other upper- or lower-limb muscle activity. Sensation was absent below T4. Patient C was a 33 year old male with 9 year C5 injury with the same pattern of paresis and paralysis but sensation was unaffected.

### Stimulation

#### Peripheral nerve stimulation

Stimuli were delivered over the left common peroneal nerve at the fibular head at a site equivalent to that which produced a twitch contraction of the pretibial flexor muscles on the right side. Stimulation over the left common peroneal nerve did not produce any contractions in the leg (though it did so in upper limb muscles, see below). The reference stimulus current was that required to evoke a reflex twitch of the contralateral digit I interphalangeal joint when delivered at ≤1 Hz. The same intensity was used to stimulate the right common peroneal nerve at the fibular head in two experiments. Stimulus pulses 0.1 ms wide were delivered (DS7AH Digitimer, UK) at frequencies from 1–100 Hz. Stimulus intensity was typically ~15-22 mA which on the right side was ~5 times motor threshold.

#### Transcranial magnetic stimulation

The motor cortex was stimulated using a circular coil (13.5 cm outside diameter) positioned over the vertex to produce a motor evoked potential (MEP) in the paretic right wrist extensor muscles (Magstim 200^2^, Magstim, UK). Current flow in the coil preferentially activated the left motor cortex. Stimulus intensity was set at 90% of stimulator output to produce as strong a corticospinal volley as possible.

#### Vibration

A 6 mm probe delivering 90 Hz vibration (Mini Vibrator, North Coast Medical, USA), was applied to muscles of the upper and lower limbs and their tendons. This stimulus was not selective for any particular mechanoreceptor, but presumably activated muscle afferents quite powerfully [25].

### Electromyography

EMG was recorded using surface electrodes from up to eight muscles in each experiment: the right and left thenar muscles; right first dorsal interosseous; right adductor pollicis; right abductor digiti minimi; right and left wrist extensors; right and left wrist flexors; right and left tibialis anterior. EMG signals were amplified 300-1000 times, bandpass filtered 16–1000 Hz, and digitized at 5 kHz. All data were collected using a 1902 amplifier, 1401 analogue-to-digital converter and Spike 2 software (CED, UK).

## Results

Here the term “interlimb reflex” is used for an involuntary contraction of upper-limb or contralateral lower limb muscle(s) in response to stimulation in a lower limb. Two types of interlimb reflex were seen. A tightly coupled interlimb reflex twitch contraction with a reproducible latency was produced by stimulation of only the left leg and appeared only in the right thumb. This interlimb reflex contraction consisted of a single twitch of EMG activity. The second form of interlimb reflex had a more variable latency and consisted of multiple discharges in one or more motor units, with activity that outlasted the triggering stimulus, and for which no other on-going stimulus was identifiable. The term “self-sustained activity” is applied to this form of interlimb reflex activity. Self-sustained activity was noted in all four limbs following stimuli to either leg.

Interlimb reflexes and self-sustained activity were observed using the criteria described here only in patient A. Similar responses could not be recorded in two healthy controls or in SCI patients B and C. In patient A the pattern of responses, both within and between limbs, was similar in the 9 experiments over 18 months, as was the stimulus intensity required to generate these responses. Unlike the tightly coupled reflex twitch of the thumb, the absolute magnitude and duration of the self-sustained muscle activation varied between recording sessions, such that mean or modal latency values provide little insight into the pathways responsible for the self-sustained discharge.

### Single stimuli

The interlimb reflex twitch at the interphalangeal joint of digit I of the right thumb had a mean latency of 69 ms following a single stimulus at 15-22 mA to the left peroneal nerve. No other activity was recorded in response to single stimuli. These twitch responses were robust, and did not “warm up” or require high-frequency stimuli, multiple stimulus bursts, multiple trials or voluntary efforts. This reflex could also be evoked from every skin site tested in the left leg including toe 2 (peroneal territory) and the lateral malleolus (sural territory). This suggests that cutaneous afferents mediated the reflex and that some afferents were able to conduct across the sciatic nerve lesion. Similar responses could be evoked by more natural mechanical stimuli to the skin of the left leg, including brushing the skin, a vibrating tuning fork and tapping the skin over the patellar tendon (see below).

### Stimulus trains

**Figure 1:**
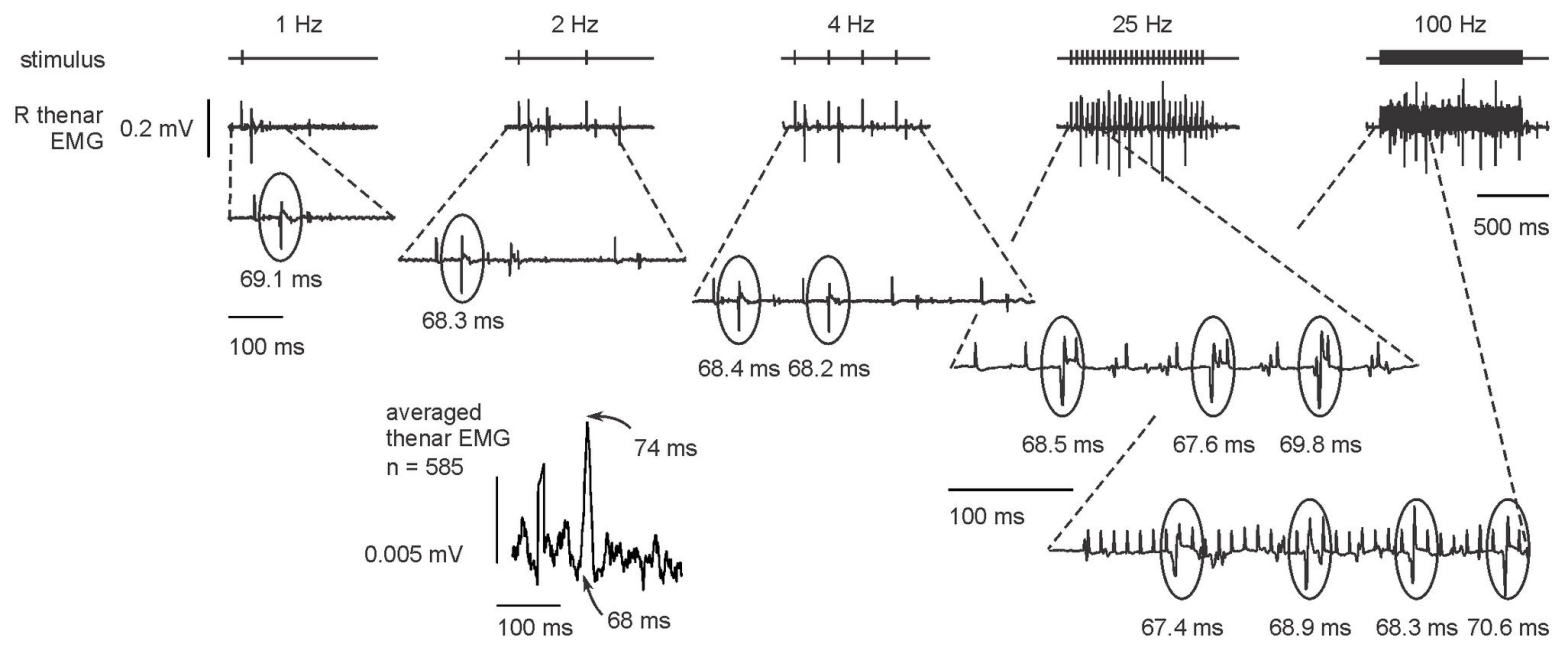
Latency of the interlimb reflex. When stimuli were applied in 1-s bursts at different frequencies, a reflex response was always seen in the right thenar EMG following the first stimulus. Each panel shows the response to stimulation for 1 s on an expanded panel where the upward-going stimulus artefact can be clearly seen in response to every stimulus, even when there is no motor response. Unrelated EMG responses can be seen, most clearly at 2 Hz where there is only one reflex response. The inset panel below shows the stimulus-triggered average EMG response from all 585 stimuli. The onset latency did not change with the rate of stimulation. The initial peak is the stimulus artefact (slightly distorted due to digitization error).

**Figure 2:**
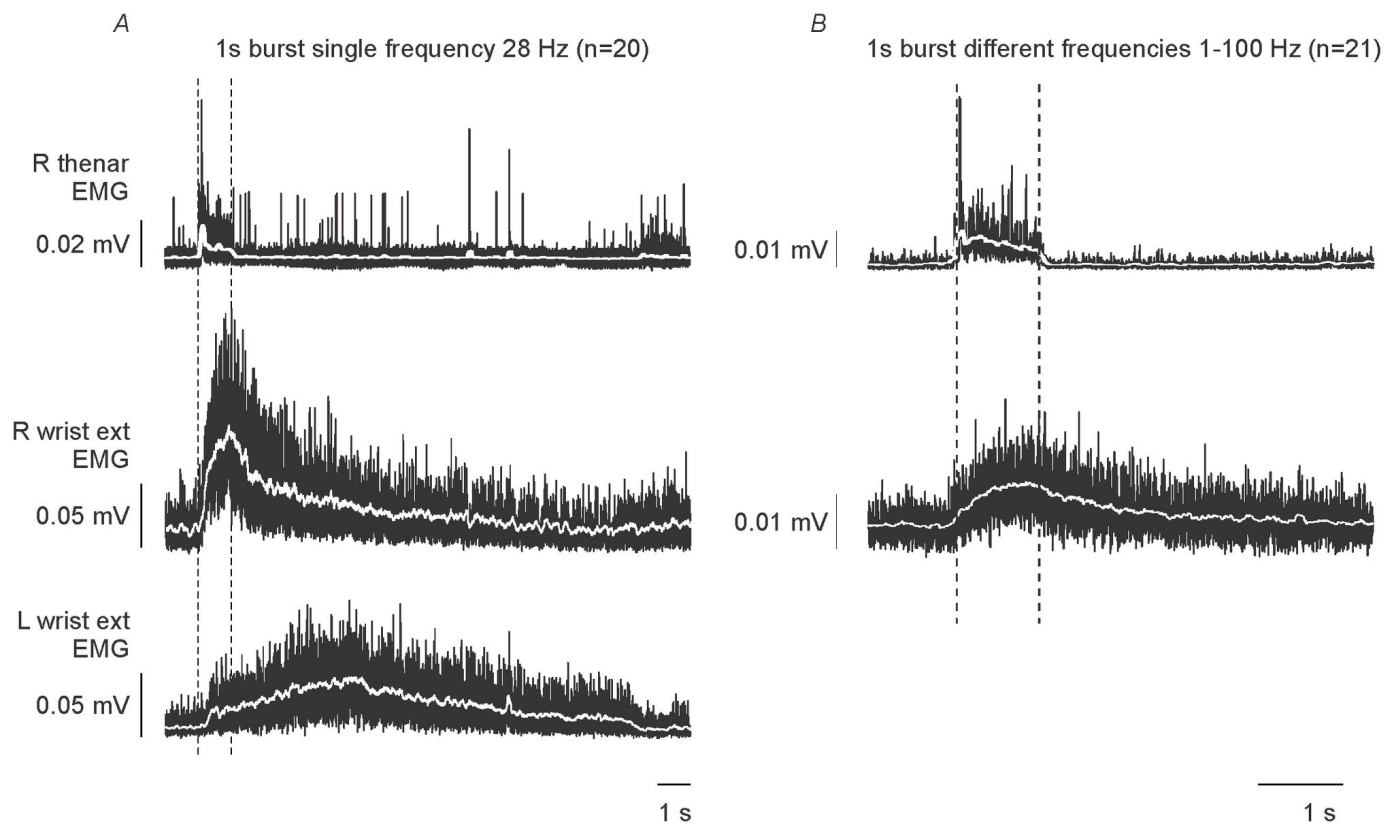
Averaged EMG response to 1-s stimulus trains at different frequencies. EMG has been rectified and smoothed with a moving 1.5-ms window. Dashed vertical lines indicate the duration of the 1-s stimulus train. A: Average of 20 trains of stimulation at 28 Hz lasting for 1 s. There were sporadic “fasciculation” potentials before and after the train but no self-sustained activity in the thenar muscles. B: Stimuli were delivered for 1 s in 21 trains at different frequencies of 1-100 Hz. Sustained activity is evident in wrist extensors but not in the thenar EMG. Data recorded in two different experiments.

Stimulus trains of 1-s duration were applied at 20 constant frequencies from 2–100 Hz. A tightly coupled thenar reflex was always present to the first stimulus of a train, and then at variable subharmonics when the rate was >1 Hz. The mean EMG latency for all experiments was 69 ms regardless of frequency (Figure 1) and discharges were not seen at shorter intervals. In addition to the tightly coupled reflex contraction of the thumb, frequencies greater than 3 Hz generated self-sustained activity in the extensors of the wrist and fingers of the right forearm but not of the intrinsic muscles of the hand. With repeated stimulation at a single frequency or with trains of different frequency, sustained activity in the paretic right wrist extensors lasted >5 s after the stimulus trains had ceased (Figure 2A,B). Similar activity occurred in the left wrist extensors, ipsilateral to the stimulus, but with slower onset and lower amplitude (Figure 2A). It is conceivable that this activity resulted indirectly through reflex connections between the upper limbs, rather than a direct left-lower-limb to left-upper-limb response.

**Figure 3:**
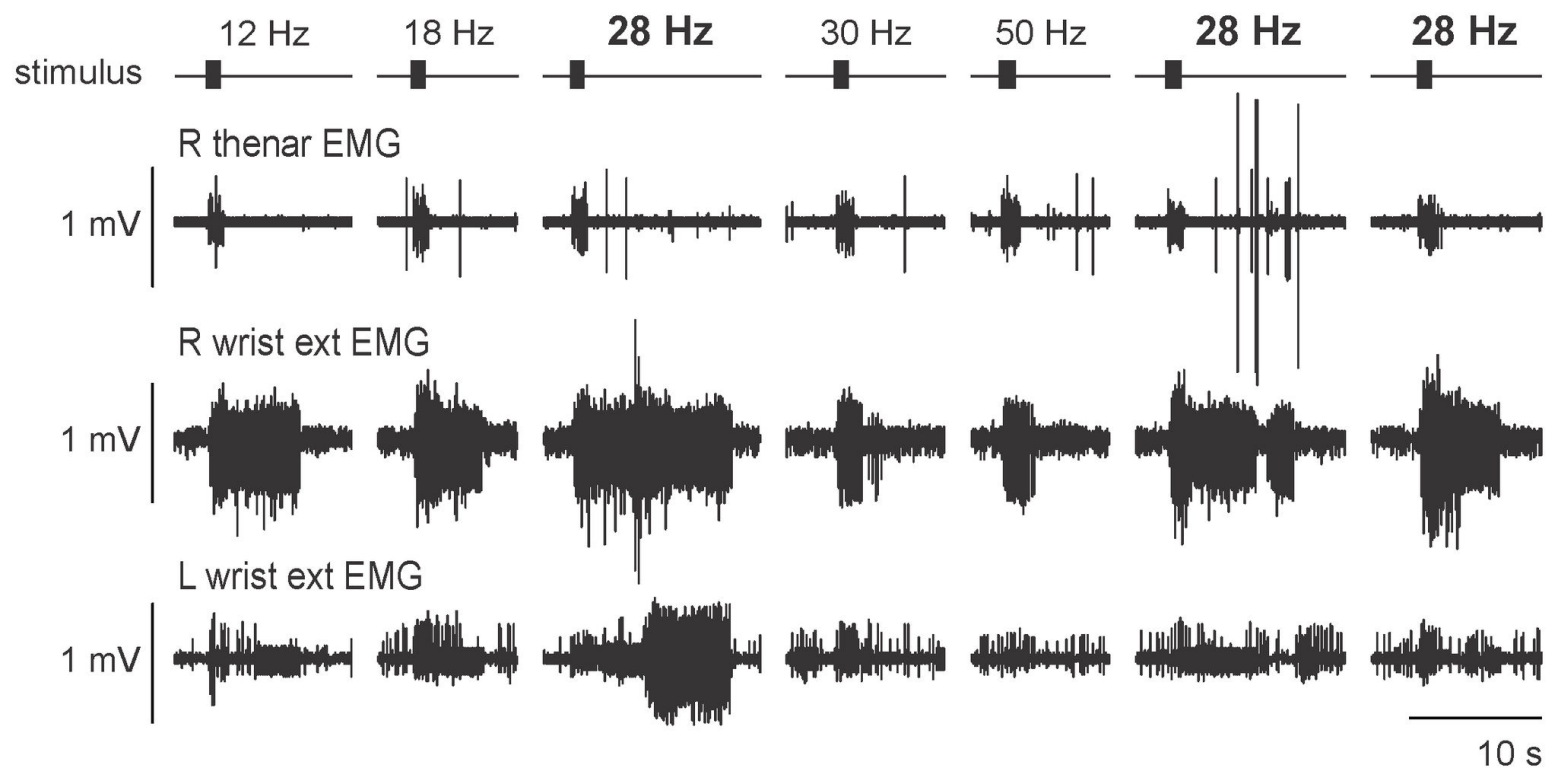
Self-sustained activity in response to 1-s trains of stimuli at different frequencies. All data were recorded within a 10-min period. Although 28 Hz produced the most consistent and longest responses, the amplitude and duration of the response, even at the same frequency varied from trial to trial. Responses were typically shorter at frequencies above 28 Hz. Data are shown in the order in which they were obtained, but some trains have been omitted.

**Figure 4:**
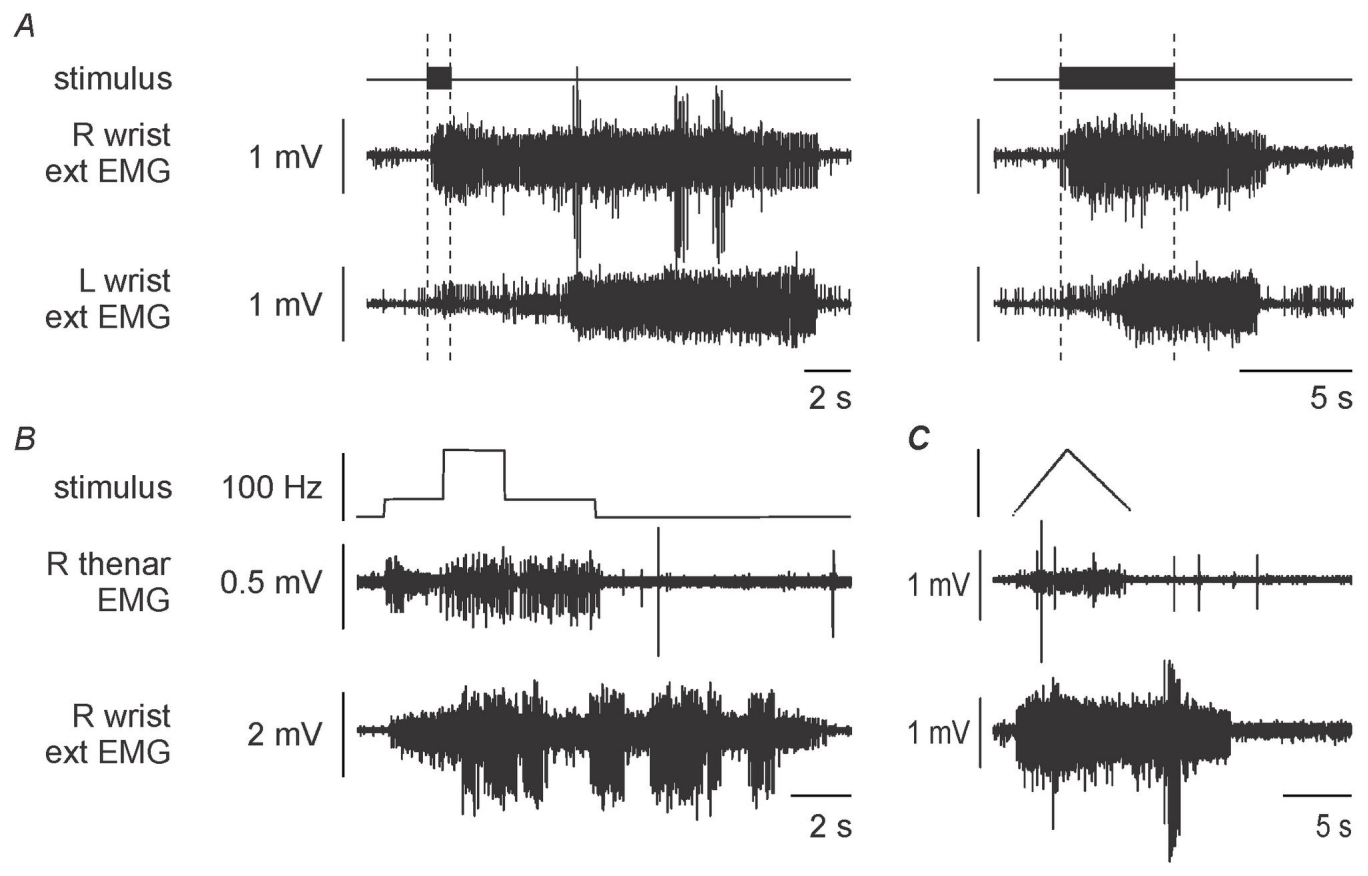
Stimulus protocols. A: Stimulus train duration. The greatest responses, both in duration and magnitude, were obtained with trains lasting 1 s. Longer train durations (right) commonly curtailed the self-sustained activity in all muscles studied. Note the consistently later response in the left wrist extensors. Dashed vertical lines indicate the duration of the stimulus trains. B–C: Responses to different stimulus patterns (see refs 14,16). Self-sustained activity was evident in the right wrist extensors with both stimulus patterns, but not in the right thenar muscles. A: 25 Hz for 2 s then 100 Hz for 2 s then 25 Hz for 3 s, C: ramp stimulus, increasing from 1 Hz to 100 Hz over 5 s and then decreasing to 1 Hz over 5 s.

The most consistent and strongest self-sustained discharge was evoked at 28 Hz (Figure 3) when the stimulus intensity was that required to evoke the thenar reflex twitch described above. However, self-sustained activity could be evoked with stimuli as low as 3 mA when the stimulus frequency was 100 Hz. This low intensity indicates that the response was not mediated by nociceptive afferents, and that temporal summation occurred within the reflex circuit. Longer stimulus trains (<5 s) did not evoke longer self-sustained activity (Figure 4A); nor did longer stimulus pulses (1 ms) within trains.

### Properties of the self-sustained activity

Evidence of “warm up” was seen with repeated stimulus trains, provided that they were not too close. For example, when five 1-s 28-Hz trains of stimuli were delivered at intervals of 1 s, continuous EMG developed in the right and left wrist extensors, and lasted >25 s, with EMG build up over the first 4.3 s. The interval between stimulus trains producing the most consistent activity in the right wrist extensors was 15 s, timed from the end of the self-sustained motor activity to the onset of the next stimulus train. As the interval was decreased to 5 s, the discharge became shorter. However, continuous activity occurred with intervals <5 s.

Self-sustained activity could be evoked by the stimulus patterns used in previous studies of “plateau-like” contractions in healthy human subjects [17] and in patients with spinal cord injury [19], including a triangular ramp of linearly changing stimulus frequencies of 1-100 Hz (Figure 4C).

Self-sustained activity was recorded in both lower limbs but was less consistent than in the upper limbs. The lower-limb activity could be triggered by stimulation of the left leg but not the right. However stimulation over the right peroneal nerve could evoke similar self-sustained activity in the upper limbs. The patient would not allow manometric recordings, thus precluding direct measures of detrusor activity but, when sustained activity was induced, the patient voided and her urine bag required emptying 1-2 times. This did not occur under non-experimental conditions. Experiments were conducted at approximately same time each day and, like most chronic SCI patients, she followed a fairly stereotypic routine. It is therefore likely that reflex contractions of the bladder accompanied the self-sustained activity.

Vibration at 90 Hz was applied to muscle or tendon to activate muscle afferents [25,26] but it neither initiated nor interrupted any response. It did not matter which muscle was vibrated, or whether vibration was applied to the muscle tendon or directly to the muscle belly. However, right wrist extension could be produced by applying a vibrating tuning fork to the skin of the left foot, or by tapping the skin over the left patellar tendon.

### Supraspinal influences

**Figure 5:**
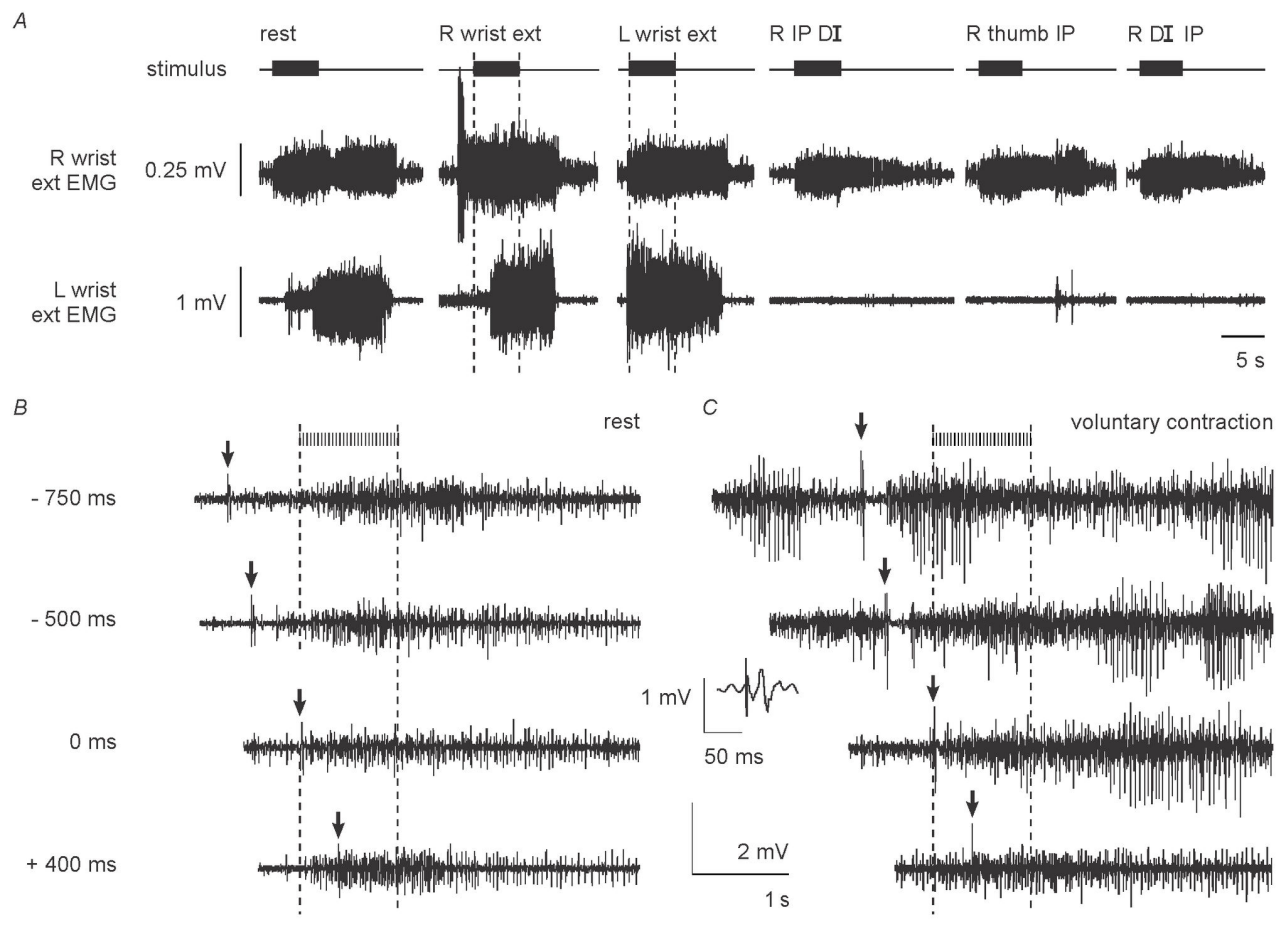
Supraspinal influences. A: The effect of voluntary effort. The subject could make weak voluntary contractions of both wrist extensors but could not grade the strength of the contractions. Isolated contraction of either the right (second panel; note that voluntary EMG precedes stimulus train) or the left (third panel; note that voluntary EMG just precedes stimulus train) wrist extensors did not greatly alter the response. When the patient attempted to contract the paralysed right thenar muscles (panels 4-6), the voluntary effort did not produce EMG in the paralysed right intrinsic muscles, but the self-sustained activity in the left wrist extensors was completely suppressed. The final three voluntary efforts were recorded in successive trials. B–C: Transcranial magnetic stimulation did not inhibit self-sustained activity in the wrist extensors. At rest (B) a small motor evoked potential was elicited in the paretic right wrist extensors by transcranial magnetic stimulation but no other changes were noted. When delivered during the stimulus train to the lower limb, there was no silent period in the train-induced EMG. The patient then performed a voluntary contraction of the wrist extensors beginning before stimuli were delivered (C). A silent period can be seen when the stimulus preceded the 1-s burst of stimuli to the left leg, but not when it was delivered during the burst. There was a period of reduced activity in the wrist extensors only when the transcranial magnetic stimulation coincided with the onset of peripheral stimulation, and not when it was delivered later during the train. The motor evoked potential is illustrated in the inset panel, preceded by the stimulus artefact.

*Voluntary contractions* of the right and left wrist extensors caused little change in the self-sustained activity (Figure 5A), but attempted contraction of the paralyzed right thenar muscles abolished the activity in the paretic left wrist extensors. When stimuli producing self-sustained activity were delivered, the subject could initiate voluntary contractions of the paretic wrist extensors but could not stop a contraction if the stimuli were delivered during the voluntary effort. Instead she had to wait for the self-sustained activity to subside. Self-sustained activity could not be initiated by voluntary effort as it can by healthy subjects [27].

Transcranial magnetic stimulation (TMS) at 90% produced a small MEP in the paretic right wrist extensor muscles at rest with a mean latency of 16 ms (Figure 5 inset), and this was immediately followed by a silent period if TMS was delivered during a voluntary contraction of the paretic muscles. No MEPs were recorded in the paralyzed thenar muscles. When TMS was delivered during and after a train of stimuli to the left leg, there was no silent period in the involuntary right wrist extensor EMG (Figure 5B). When TMS and stimuli to the left leg were delivered during a voluntary contraction of the right wrist extensors (Figure 5C), there was a silent period when the TMS preceded the peripheral stimulation. If TMS coincided with the first pulse, the self-sustained activity was reduced for 88 ms but there was no silence (panel 3 in Figure 5C). The duration of the silent period produced during a voluntary contraction ranged from 197 to 227 ms with a mean of 217 ms. These findings indicate that the EMG activity triggered by (or associated with) the interlimb reflex was not modulated as is a voluntary contraction, even though mechanisms sufficient to produce a silent period remained intact. This finding favors the view that the self-sustained activity involved intrinsic spinal cord mechanisms that were operating in a relatively autonomous mode.

## Discussion

The findings in this exceptional case indicate that plastic changes occur in spinal circuitry after SCI and provide strong evidence for a functionally important ascending propriospinal pathway in humans. We have documented a simple interlimb reflex with a 1:1 connection via a long propriospinal pathway, in which single low-threshold electrical stimuli applied to the lower limb resulted in a robust reflex twitch contraction in the contralateral thumb. In addition we provide the first report of robust self-sustained activity initiated by the low-threshold cutaneous afferents, possibly mediated by PICs in upper limb motoneurons, as discussed below. Neither interlimb reflexes [10,20,29,30] nor self-sustained activity [19,20] are unique after spinal cord injury. Rather it is the pattern encountered in this subject that is unique.

The self-sustained interlimb reflex contractions required trains of weak electrical stimuli, and could be recorded in all four limbs. However the strongest responses were recorded in the right wrist extensor muscles after stimulation to the left leg, a pattern different to the stronger ipsilateral projection documented for ascending propriospinal projections in healthy subjects [8,9]. This case emphasizes the role of altered neuronal properties in the hyperreflexia that accompanies SCI, and we conclude that PICs can be triggered in motoneurones in segments remote from the level of the afferent input. Whether the connections seen here result from unmasking of relatively weak pathways present in healthy subjects or require sprouting and the creation of new spinal connections is discussed below.

Another novel finding of this study is the distribution of the self-sustained activity. In previous studies on healthy subjects, self-sustained activity was evoked in the muscle being stimulated [17] or voluntarily contracted [28]. Similarly, after spinal cord injury self-sustained activity has been evoked in the muscle being electrically stimulated [19] or that in which muscle spasms were induced [19,24]. Self-sustained activity has previously been reported in adductor pollicis in a SCI patient in response to ice applied to the contralateral lower limb [20]. This activity was presumably mediated by small-diameter A-δ fibers whereas the activity in the present patient was presumably mediated by low-threshold large afferents. This supports the view that tactile afferents can produce involuntary reflex activity and trigger spasms in SCI patients.

The sustained motor activity seen in this subject is exceptional, and was not seen in other patients with the same stimulation protocols. That it can occur illustrates the potential for plastic changes in spinal circuitry to produce adaptive (and maladaptive) responses in SCI. This view is reinforced by the findings of Calancie and colleagues [10,20,29,30] which, though different, also represent behavioral features that emerged after injury, presumably in response to it.

### Were the interlimb reflexes transmitted through a propriospinal pathway?

Interlimb reflexes have previously been studied in SCI subjects only by Calancie and colleagues [10,20,29,30], and not surprisingly, there are similarities and some differences in our findings. The reflex in Figure 1 is strong evidence for a functional long propriospinal connection [4,5,31]. A transcortical pathway is unlikely because it would require an intact afferent projection from the legs across the spinal cord lesion and then a retained efferent limb back into damaged spinal cord segments, sufficient to activate the paralyzed thenar muscles when volitional and TMS-induced corticospinal volleys could not. The absence of a silent period to TMS (Figure 5C) also argues against a supraspinal loop for the self-sustained activity. Instead the activity was presumably generated by circuits intrinsic to the spinal cord, capable of operating in an autonomous mode, once triggered.

The 69 ms latency for the reflex twitch in the right thenar muscles did not change with stimulus frequency or intensity, and this suggests an oligosynaptic pathway with a direct projection. The response could be generated by low-threshold, presumably tactile afferents and did not require convergence with smaller-diameter thermal or nociceptive afferents. The 69-ms latency is consistent with the slow conduction velocity of human propriospinal circuits: estimated to be 5 ms^-1^ [32], and 13 ms^-1^ [31]. It is slower than reported for descending propriospinal pathways in humans [33], and some 20 ms or more slower than the shortest latencies for ascending pathways in the studies of Calancie and colleagues [10,20,29,30]. In these reports the latency varied with stimulus frequency, intensity and pulse width, suggesting a more complex and a less secure pathway, possibly with temporal summation and convergence between different afferent species. Such differences are not surprising: it is likely that there are multiple spinal pathways linking the upper and lower limbs, ascending and descending, some relatively direct, others relayed through a number of spinal interneurones.

Evidence for the role of long propriospinal pathways in interlimb co-ordination comes from studies in which coordinated locomotion was abolished by serial spinal cord lesions in the cat [2,34], and pharmacological lesions in the rat [35]. The anatomical organization of these pathways is similar in the cat and human [36,37]. The functional importance of descending long propriospinal pathway in humans has been emphasized in complete spinal cord-injured patients in whom noxious stimulation of thoracic, lumbar and sacral dermatomes differentially modulated the Achilles tendon jerk reflex [31]. Previous studies of ascending propriospinal connections in healthy humans have reported stronger ipsilateral than contralateral responses [8], and these responses have been suggested to have a functional role in recovering from locomotor perturbations [8,9]. This pattern of interlimb reflex is consistent with a pacing gait, and could be demonstrated in this patient with stimulation of the right leg. However, the strongest response in this patient was in the contralateral upper limb, and this is the pattern of a walking gait. A bidirectional propriospinal linkage between the upper and lower limbs could be important in retraining locomotion in paraparetic patients, as proposed by Dietz [1].

### Do the interlimb reflex connections represent new connections due to sprouting of axons or unmasking of pathways normally present?

Calancie and colleagues have attributed the development of interlimb connections in SCI to “new growth (regenerative sprouting) in the spinal cord caudal to a severe injury” (p. 1150 in ref. [29], see also ref. [20]), and have argued that the strengthening of these reflex responses with time was consistent with on-going synaptic plasticity [10]. These are important findings because they demonstrate the ability of the damaged spinal cord to generate adaptive responses long after the injury. However it is debatable whether such findings necessarily imply the development of new connections rather than the strengthening of previously inapparent weak propriospinal connections. Collateral sprouting would presumably contribute to both alternatives. We favor the more parsimonious second alternative, in part because the pattern is consistent with propriospinal pathways seen in the cat, and because the slow development can be explained by both sprouting and adaptive changes in sensitivity to endogenous monoamines (see below).

### The basis of the self-sustained activity

PICs driven by descending monoaminergic inputs from raphe nuclei in the brainstem underpin self-sustained activity in intact animals [38]. These projections are distributed widely in the spinal cord [39], and their interruption is highly probable in our patient. However, the appearance of constitutively active 5-HT_2_ receptors in spinally transected animals can explain the emergence of self-sustained activity after SCI [40]. In the present patient, stimulation for only 1 s caused sustained activity in the contralateral wrist extensors (Figure 3). The patient could not terminate the activity, and voluntary contraction of the paretic wrist extensors did not substantially alter the response (Figure 5A), though attempted voluntary contraction of the paralyzed right thenar muscles abolished it in the paretic left wrist extensors. This pattern is reminiscent of that in animal preparations due to PICs [41], which can be terminated by an injected inhibitory current. A comparable sustained motoneurone discharge can be evoked in healthy human subjects by muscle tendon vibration during voluntary contractions [16], electrical stimulation of relaxed muscles [17] and during voluntary efforts [27]. This stimulus-induced activity can be terminated spontaneously (i.e., without inhibitory inputs) or by voluntary relaxation. Similar protocols produce self-sustained activity in spinal-cord injured patients with both complete and incomplete injuries [19]. However as in the present study, self-sustained activity in spinal-cord injured patients cannot be terminated voluntarily despite, on some occasions, significant discomfort (P. Nickolls, personal communication).

In previous studies on healthy and SCI humans, the self-sustained activity was thought to be mediated predominantly by muscle spindle afferents, not cutaneous afferents [17,19]. In the present experiments stimulation over the left peroneal nerve did not produce any contraction of the pretibial flexor muscles, and neither muscle nor tendon vibration in either lower limb produced self-sustained activity. However, such activity could be elicited by stimulation activating mainly cutaneous afferents, and at current levels as low as 3 mA, suggesting low-threshold afferents, not nociceptive. Interlimb reflexes can be mediated by cutaneous afferents [20], and the same inputs that produced the interlimb reflex in the thenar muscles in this patient also triggered the self-sustained activity.

Studies in hemiplegic patients suggest that sustained motoneurone discharges can occur through other as-yet unclarified mechanisms, largely independent of PICs [42]. However there is now substantial literature indicating that PICs are responsible for self-sustained activity in the spinal rat, and there is strongly suggestive evidence that this is also so in SCI humans [21–24,43]. The strongest human evidence comes from paired motor unit recordings of Gorassini and colleagues [27], though it is worth noting that this evidence has recently been questioned: in the cat the lower firing rates of motoneurones at de-recruitment than at recruitment can be partly explained by prolongation of the post-spike afterhyperpolarization (AHP) following spike trains [44]. Nevertheless, the weight of evidence favors an important role of PICs in the spinal hyperexcitability of SCI, and it is important that the properties seen in our patient are those expected given the properties and consequences of PICs in motoneurones.

### Clinical implications

The findings in this exceptional case provide strong evidence for the existence and function of ascending long propriospinal pathways in humans. There is mounting evidence that long propriospinal pathways help co-ordinate movements of the upper and lower limbs during bipedal locomotion, as they do in quadrupeds. Given the hypothesised importance of propriospinal neurones in interlimb co-ordination, it is remarkable that they have not been studied more extensively. Our results confirm many findings on interlimb reflexes in studies published before the role of PICs in driving a sustained motoneurone discharge was appreciated [10,20,29,30]. Our findings suggest that PICs may be triggered by inputs that arise remote from the motoneurone pool and, specifically, that inputs from one lower limb can trigger sustained activation of motoneurones supplying upper-limb muscles. We have demonstrated that the pattern of stimulation is not critical in generating sustained activity in this subject, and that these responses can be triggered by low-threshold, cutaneous inputs to the spinal cord. Finally, these data provide additional evidence that PICs may underlie the hyperexcitability of the spinal reflex circuitry in SCI, complementing previous reports [21].

Other than the responses reported by Calancie (and presumably mediated by A-δ afferents) [20], self-sustained activity has been reported after spinal cord injury only in the stimulated limb [19,24]. The findings reported here are uncommon, if not unique, and we were unable to reproduce them using the same stimulation protocols in other patients with spinal cord injury. The stability of these responses over time suggests that they had become anatomically relevant and that if such plasticity could be controlled and directed appropriately, it might enhance motor function after spinal cord injury by strengthening nascent pathways. While we have documented only an ascending propriospinal circuit, a functional bidirectional pathway could be of value in the restoration of gait in chronic spinal-cord injury, the ascending pathway perhaps contributing to balance control, as in healthy subjects [8,9].
